# Socio-Emotional Concern Dynamics in a Model of Real-Time Dyadic Interaction: Parent-Child Play in Autism

**DOI:** 10.3389/fpsyg.2019.01635

**Published:** 2019-07-16

**Authors:** Casper Hesp, Henderien W. Steenbeek, Paul L. C. van Geert

**Affiliations:** ^1^Department of Developmental Psychology, University of Groningen, Groningen, Netherlands; ^2^Amsterdam Brain and Cognition, University of Amsterdam, Amsterdam, Netherlands; ^3^Department of Psychology, University of Amsterdam, Amsterdam, Netherlands

**Keywords:** autism, dyadic play, social skills, play initiation, child-parent, dynamical model, complexity

## Abstract

We used a validated agent-based model—Socio-Emotional CONcern DynamicS (SECONDS)—to model real-time playful interaction between a child diagnosed with Autism Spectrum Disorders (ASD) and its parent. SECONDS provides a real-time (second-by-second) virtual environment that could be used for clinical trials and testing process-oriented explanations of ASD symptomatology. We conducted numerical experiments with SECONDS (1) for internal model validation comparing two parental behavioral strategies for stimulating social development in ASD (play-centered vs. initiative-centered) and (2) for empirical case-based model validation. We compared 2,000 simulated play sessions of two particular dyads with (second-by-second) time-series observations within 29 play sessions of a real parent-child dyad with ASD on six variables related to maintaining and initiating play. Overall, both simulated dyads provided a better fit to the observed dyad than reference null distributions. Given the idiosyncratic behaviors expected in ASD, the observed correspondence is non-trivial. Our results demonstrate the applicability of SECONDS to parent-child dyads in ASD. In the future, SECONDS could help design interventions for parental care in ASD.

## 1. Introduction

“*(…) if you give a man a fish he is hungry again in an hour*.

If you teach him to catch a fish you do him a good turn.”

Mrs. Dymond, by Ritchie (1885, p.342)

Children who are suffering from moderate to severe forms of Autism Spectrum Disorders (ASD) are oftentimes caught in a vicious circle: their difficulties acquiring social skills deprive them of further opportunities to develop these skills. Over the past few decades, many researchers have aimed to increase therapeutic benefits for these children (for a review, see Walton and Ingersoll, 2013). The question remains how to approach the complications that arise in real-time social interactions between children with ASD and their surroundings and how to minimize cumulative negative effects on social development. ASD is a class of neurodevelopmental disorders where children typically experience socio-emotional difficulties when interacting and communicating with others (American Psychiatric Association, 2013; Yenkoyan et al., 2017; Sharma et al., 2018; Wadsworth et al., 2018). The common approach of linear modeling cannot capture the reciprocal and iterative causal influences characteristic of these ongoing interactions—including those between a child and its caregiver. A growing number of researchers advocate the application of non-linear dynamics (“the complexity approach”) to social and developmental psychology (e.g., Schlesinger and Parisi, 2001; Smith and Conrey, 2007; van Geert, 2011). In particular, agent-based models have been successfully employed to translate psychological theory into specific mechanisms of action for the agents in question. These models can be directly compared with the target system to directly test their plausibility (as we do in section 2.5; for a review on agent-based-model validation, see Gräbner, 2018). As such, agent-based modeling is a crucial tool that connects psychological theories to complex real-life examples. This research allows us to demonstrate how dynamical interactions between a child and its caregiver help to understand the idiosyncratic phenomenology associated with ASD (Waterhouse, 2013; Vivanti et al., 2014; Byrge et al., 2015; Hahamy et al., 2015).

A detailed understanding of the dynamics involved in social interactions of children with ASD is necessary for designing therapeutic interventions that foster their social development. Since a predominant component of a child's social interactions involves their caregivers, this relationship warrants special attention. We need to empower caregivers of children with ASD to engage these children in ways that work effectively toward key developmental milestones. By taking into account idiosyncratic and atypical socio-emotional functioning (Vivanti et al., 2014; Hahamy et al., 2015), we can provide caregivers with instructions that are carefully tailored to their particular child. The saying referenced above highlights the cumulative and non-linear nature of social impairments in children with ASD and—by extension—the clinical relevance of our complexity-oriented research. Of course, the experience of playing together (“give a man a fish”) is valuable in itself for the development of social skills inherent in play. It is known to have therapeutic effects on children with ASD (see the evidence summarized by Hull, 2015). However, it is also necessary for a child to learn how to initiate play (“teach him how to catch a fish”). Social initiation has been shown to be a pivotal response class for children with autism (Koegel and Koegel, 2006): improvement on this skill results in measurable overall improvements of the child's development. Unfortunately, a caregiver who always takes the initiative to play together will deprive their child from opportunities to practice play initiation. Balancing these two learning goals (playing together and play initiation) will therefore be a recurring theme throughout this article.

In sum, we adopt the complexity approach as we construct an agent-based model of parent-child play in the case of a child with severe ASD. Our primary goal is to translate psychological theory to empirical and clinical work in ASD, by modeling the micro-dynamics of dyadic playful interaction.

### 1.1. A Complexity-Based Approach to Developmental Psychology

“*The whole is other than the sum of its parts.”*

Koffka (1999, p.176)

Over the last few decades, complexity-based approaches have become increasingly prevalent in social and developmental psychology (Thelen and Smith, 1994; Schlesinger and Parisi, 2001; Smith and Conrey, 2007; Spencer et al., 2011; van Geert, 2011). This research has focused on the ways in which socio-emotional developmental trajectories emerge from interactions between components: a child and its social environment. Through self-organization, such a dynamical system can exhibit behaviors that are not reducible to any of its sub-systems—the child, its parents, siblings, et cetera. If we focus on one of these sub-systems—for example, the child—we find that its socio-emotional functioning in turn relies on interactions between sub-personal components, such as socio-emotional concerns, drives, and appraisals. Looking at child development from this perspective demonstrates the futility of the nature-vs.-nurture debate. The complexity perspective allows us to model the ongoing transactional effects of nature on nurture and vice versa. A number of recent analyses (e.g., Kunnen et al., 2012) addressed the interconnectedness between different levels under study in emotional development (internal, individual, dyadic, and group-wide) on various timescales associated with perception, learning, and development. While the behavior of children inherently depends on their social context, it tends to be treated merely as a set of additional variables by mainstream statistical analyses in developmental psychology. In contrast, dynamic systems approaches place a stronger emphasis on the reciprocal dependency between the child and other social components of the system (such as another child or parent). In this way, agent-based models have been successfully employed to characterize emergent behaviors (see Gräbner, 2018, for a recent review).

When modeling the time course of processes occurring within and between individuals, dynamic treatments outperform more common approaches that focus on statistical relationships between population-based, inter-individual distributions of two or more variables. The standard practice of psychology involves using statistical models to explain inter-individual variability (even when applied to time-series of individuals) and derive conclusions about individuals, which are treated as specific cases of the general models. Group-to-individual generalizations are only appropriate if data on individuals (over time) asymptotically follow the same distribution as data across individuals in the population (at any point in time). For more in-depth discussions on the statistical assumption of *ergodicity*, we refer to Molenaar (2004), Valsiner et al. (2009), and Toomela and Valsiner (2010). According to large swathes of empirical and theoretical work, this assumption is invalid for most measurable variables of psychological processes occurring within and between individuals (see, e.g. Molenaar, 2004; Kelderman and Molenaar, 2007; Molenaar and Campbell, 2009; Hamaker, 2012; Koopmans, 2015; Beltz et al., 2016; Hamaker and Wichers, 2017; Fisher et al., 2018). In fact, group statistics rarely represent individual cases and processes (a misconception also referred to as the ecological fallacy). For example, the average visiting frequencies at theme and amusement parks are structurally different from the visiting frequencies of individual visitors and families, as the latter are governed by idiosyncratic preferences^1^. Sample-based statistics are therefore likely to occlude interaction processes in parent-child dyads, especially given the idiosyncrasy of ASD (e.g., Vivanti et al., 2014; Hahamy et al., 2015). In contrast, agent-based models focus on the reciprocal causal relationships that characterize processes within a particular child and its environment, both on the short-term (e.g., real-time interactions) and on the long-term (e.g., development). The interaction across time scales is an important direction for future research, because it allows for modeling long-term therapeutic outcomes based on specific parental strategies on the time scale of individual sessions.

### 1.2. A Matter of SECONDS: Agent-Based Modeling of Socio-Emotional Concern Dynamics

A logical starting point for understanding social interaction is that of the dyad. We employed a validated agent-based model of socio-emotional concern dynamics, which has been developed and successfully applied to several kinds of dyads (e.g., child-peer play and student-teacher coupling; Steenbeek and van Geert, 2005, 2008, 2013; Schuhmacher et al., 2014). For future reference, and with consent from the original authors of this model (the co-authors on this paper), we give this agent-based model the acronym *SECONDS* (Socio-Emotional CONcern DynamicS). Steenbeek and van Geert (2013) have used it to model dynamical scaffolding in teacher-student dyads in order to help develop teaching strategies for finding the optimal “scaffolding distance” of learning (i.e., the difference in difficulty level between the teacher's explanation and the current level of understanding of the child). SECONDS involves dynamic, iterative relationships between socio-emotional concerns, drives, appraisals, and behaviors of each member of the dyad.

As implied by its acronym, SECONDS generates real-time interaction data on timescales of seconds. It has been shown to produce plausible real-time data of playful dyadic interaction between children (Steenbeek and van Geert, 2005, 2008; Steenbeek et al., 2014). To our knowledge, there are no applied alternatives available for this type of dyadic interaction other than SECONDS. Besides observational validation, the plausibility of agent-based models like SECONDS also hinges on the theoretical considerations motivating the constituent components and their connections. The methodological considerations underlying the validation of dynamical models forms a recurrent theme throughout this article. The criteria for model validation in process-oriented dynamical modeling approaches differ significantly from those of standardized statistical methods and verbal theorizing typical in the field of developmental psychology. Given that our core goal in conducting this research has been to translate from theory to practice, the concept of plausible representation and other methodological concepts associated with model-validation in dynamic systems modeling are unpacked in sections 2 and 4, with a special focus on our particular application in developmental psychology.

Previous empirical validation of SECONDS involved the context of child play between peers and the interaction between a teacher and a child in an instructional setting. In principle, it can be adjusted to model impaired socio-emotional capacities, with potential applications for the design of therapeutic interventions. With this translational research, we aimed to explore this possibility, theoretically and empirically. We therefore extended SECONDS to playful interaction between a parent and a child with ASD.

## 2. Materials and Methods

### 2.1. SECONDS: An Agent-Based Model of Dyadic Interaction

**Figure 1:**
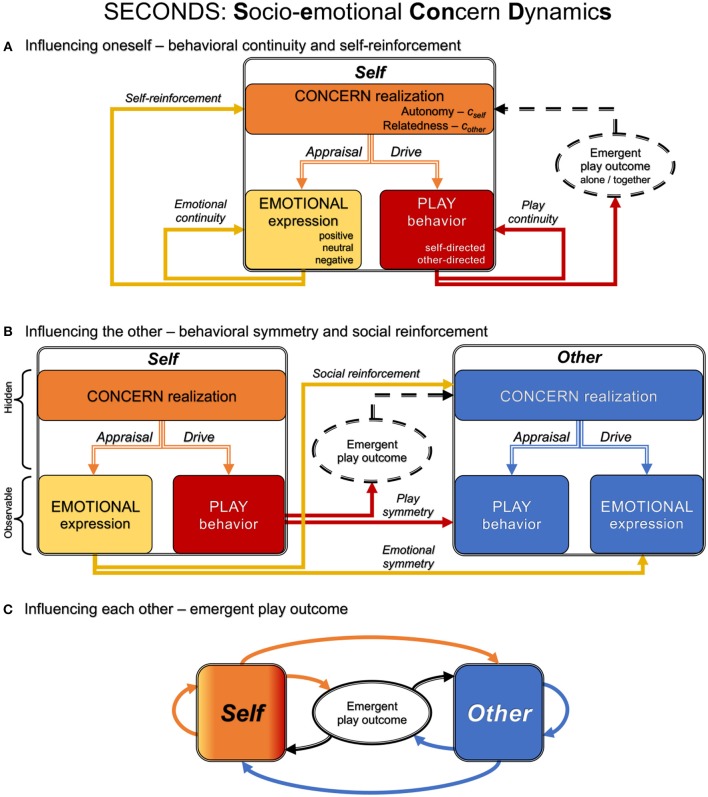
These panels illustrate Socio-emotional Concern Dynamics (SECONDS) from the perspective of one agent (*Self*) in playful interaction with another agent (*Other*). For clarity, we first decompose SECONDS into influences of the agent on itself (*Self* ⇒ *Self*) in **(A)**, and on the other agent (*Self* ⇒ *Other*) in **(B)**. Finally, in **(C)**, we illustrate the full model of how their mutual influences give rise to the emergent play outcome (*Self* ⇔ *Other*). **(A)** The agent monitors the realization of its socio-emotional concerns of autonomy and relatedness (*c*_*self*_, *c*_*other*_; orange box). The degree of realization of the agent's concerns produces corresponding appraisals and drives, which influence its emotional expressions (positive, neutral, or negative; yellow box) and play behaviors (self- or other-directed; red box), respectively. Emotional expressions tend to persist into the future (emotional continuity) and to modify the agent's own concerns (self-reinforcement). Play behaviors also tend to persist into the future (play continuity) and contribute to playing alone or together in the emergent play outcome (dashed ellipse), thus impacting the agent's concern realization (dashed black arrow). **(B)** We emphasize a crucial distinction between external active states, which are observable to the other (*Other*; blue), and internal states of the agent, which are hidden from the other. Emotional expressions tend to be mirrored by the other (emotional symmetry) and modify the other's concerns (social reinforcement). Play behaviors tend to be mirrored by the other (play symmetry) and contribute to the emergent play outcome (dashed ellipse), thus impacting the other agent's concern realization (dashed black arrow). **(C)** The full picture of how the recurrent play dynamics between *Self* (left; orange box and arrows) and *Other* (right; blue box and arrows) give rise to the emergent play outcome (middle; solid black ellipse and arrows). In panel c, the emergent play outcome is fully specified (indicated with solid lines), while it is only partially specified in panels a and b (indicated with dashed lines). See Table 1 for a more technical summary of SECONDS.

As an agent-based model, SECONDS (Steenbeek and van Geert, 2005, 2008) entails a model of agency. Gräbner (2018) provides a recent and comprehensive review of the methodology of agent-based models and their empirical validation. Such models focus on the mechanism of action, where a mechanism is defined as a system of connected components. SECONDS specifies the components and connections necessary to describe how an agent influences itself and how agents influence one another, as primarily inspired by the emotion theory proposed by Frijda (1986). It characterizes agency as an interaction between socio-emotional concerns, drives, appraisals, and behaviors of each agent. Together, these components and their interactions generate an emergent sequence of events (i.e., a discontinuous time series). Direct relationships between these components define second-to-second interactions while influences across the session are incorporated via a memory component. We introduce SECONDS by describing the influences within an agent (*Self* ⇒ *Self*), between agents (*Self* ⇒ *Other*), and emergent influences (*Self* ⇔ *Other*), as shown in Figure 1. In this context, we refer to the agent as an child or a parent, but in principle SECONDS applies to any agent whose mechanism of activity depends on socio-emotional concerns. In Table 1, we also provide a technical summary of SECONDS that highlights the relevant parameters (*a*_1−9_). The size of time steps was set to be four seconds, corresponding with the observational resolution of the coding system. Where applicable, we also added footnotes concerning associated concepts in dynamic systems theory.

**Table 1:** A simplified technical summary listing the coupled equations of Socio-emotional Concern Dynamics (SECONDS) between *Agent x* and *Agent y*, described by individual variables *x*_1−5_ and *y*_1−5_, respectively, and joint variable *z*.

**Variables**	***Agent x, Agent y***	**Partial dependencies for** ***Agent x***				**Full dependencies for *Agent x***
		Initial concerns	[*c*_*self*_, *c*_*other*_]	=	*x*_1,_*t*__0__	
Concerns	*x*_1_, *y*_1_	Continuity	*x*_1, *t*_	→	*x*_1, *t*+1_	Δ*x*_1, *t*_ = *f*_1_(*x*_3, *t*_, *x*_4, *t*_, *y*_4, *t*_)
		Self-reinforcement	*x*_3, *t*_·*x*_4, *t*_	$\overset{a_{1}}{\underset{\;}{\to }}$	*x*_1, *t*+1_	
		Social reinforcement	*x*_3, *t*_·*y*_4, *t*_	$\overset{a_{2}}{\underset{\;}{\to }}$	*x*_1, *t*+1_	
Concern realization	*x*_2_, *y*_2_		*x*_1, *t*_−*x*_5, *t*_/*t*	$\overset{a_{3}}{\underset{\;}{\to }}$	*x*_2, *t*_	*x*_2, *t*_ = *f*_2_(*x*_1, *t*_, *x*_5, *t*_)+ω
		Drives	*x*_2, *t*_	$\overset{a_{4}}{\underset{\;}{\to }}$	*x*_3, *t*+1_	
Play behavior	*x*_3_, *y*_3_	Continuity	*x*_3, *t*_	$\overset{a_{5}}{\underset{\;}{\to }}$	*x*_3, *t*+1_	*x*_3, *t*+1_ = *f*_3_(*x*_2, *t*_, *x*_3, *t*_, *y*_3, *t*_)
		Symmetry	*y*_3, *t*_	$\overset{a_{6}}{\underset{\;}{\to }}$	*x*_3, *t*+1_	
		Appraisals	*x*_2, *t*_	$\overset{a_{7}}{\underset{\;}{\to }}$	*x*_4, *t*+1_	
Emotional expression	*x*_4_, *y*_4_	Continuity	*x*_4, *t*_	$\overset{a_{8}}{\underset{\;}{\to }}$	*x*_4, *t*+1_	*x*_4, *t*+1_ = *f*_4_(*x*_2, *t*_, *x*_4, *t*_, *y*_3, *t*_)
		Symmetry	*y*_4, *t*_	$\overset{a_{9}}{\underset{\;}{\to }}$	*x*_4, *t*+1_	
Memory	*x*_5_, *y*_5_	Retention	*x*_5, *t*_	→	*x*_5, *t*+1_	*x*_5, *t*+1_ = *x*_5, *t*_+*z*_*t*_
		Storage	*z*_*t*_	→	*x*_5, *t*+1_	
Play outcome	*z*	Emergence	*x*_3, *t*_·*y*_3, *t*_	→	*z*_*t*_	*z*_*t*_ = AND(*x*_3, *t*_, *y*_3, *t*_)

*The individual variables are Concerns (continuous vectors x_1_ and y_1_, length two), Concern realization (continuous vectors x_2_ and y_2_, length two), Play behavior (binary vectors x_3_ and y_3_, length two), Emotional expression (categorical x_4_ and y_4_, taking values [-1,0,1]), and Memory (count vectors x_5_ and y_5_, length two). The joint variable is the Play outcome (binary vector z, length two). For each of the variables of Agent x, we list the partial dependencies (third column) and full dependencies (fourth column). Partial dependencies involve a single variable (e.g., Drives involve x_2_), a multiplicative interaction between two variables (e.g., Social reinforcement involves x_3_ and y_4_), or the difference between two variables (Concern realization compares x_5_ and x_1_). Arrows with a_1−9_ indicate relevant parameters of SECONDS that modulate the connection strengths of the corresponding dependencies. For example, the impact of Social reinforcement on x_1_ is regulated by a_1_, et cetera. Full dependencies are listed as explicit simple functions (e.g., z is the AND function of x_3_ and y_3_) or more complex functions omitted for readability (f_1−4_). Every time step, Concerns change by Δx_1_ = f_1_(x_3_, x_4_, y_4_). Concern realization is defined by a difference function f_2_(x_1_, x_5_) plus random variability ω, which generates the stochastic properties of SECONDS. Categorical outcomes of Play behavior and Emotional expression are generated from continuous input using step functions f_3_ and f_4_. All the equivalent relations for Agent y can be obtained by switching variables x_1−5_ and y_1−5_. See Figure 1 for a graphical illustration of SECONDS*.

#### 2.1.1. Influencing Oneself

Agency involves specific goals or motives, which can be derived from the *concerns*^2^ of an agent combined with its context. The current implementation of SECONDS focuses on the tension between socio-emotional concerns of relatedness vs. autonomy. In the work of Frijda (1986), the relatedness concern pertains to “identity striving” which entails a focus on closeness and connectedness. In SECONDS, initial concerns are represented by parameters that indicate to which extent the child experiences relatedness and autonomy (*c*_*other*_, *c*_*self*_) as rewarding. Together with a specific context such as dyadic play, concerns specify which immediate actions are relevant: self- or other-directed *play behavior*^3^. For a child in such a dyad, self-directed play is mostly relevant to its autonomy concern and other-directed play to its relatedness concern. Since these are (approximately) mutually exclusive, the concerns in effect define the proportion between these behaviors that is experienced as optimally rewarding by the child (i.e., *c*_*other*_ + *c*_*self*_ = 1.00)^4^. While concerns are relatively stable characteristics of a child (i.e., initial conditions for each simulated interaction), they are also influenced by real-time changes in the emotional content of an interaction through self- and social reinforcement (parameters *a*_1_, *a*_2_ in Table 1; explained below). As for the initial values of these concerns in the context of dyadic play, typically developing children have been found to be more concerned with relatedness than with autonomy (*c*_*other*_ ≈ 0.70, *c*_*self*_ ≈ 0.30; Steenbeek and van Geert, 2005, 2008).

Aiming for an optimally rewarding balance, the child infers the extent to which its socio-emotional concerns are currently being met or realized (see Figure 1A). *Concern realization* is the perceived match between outcomes in the current session (salient in memory) and preferred session outcomes (set by the child's concerns)^5^. The sensitivity of concern realization is modulated by *a*_3_ – it effectively summarizes the situation from the perspective of the child: “how am I doing?” The child's reactions to that evaluation consist of behavioral *drives* and *emotional appraisals*, respectively (*a*_4_, *a*_7_ in Table 1)^6^. If a child infers its preferred outcomes are being realized, no additional drive is required and positive emotional appraisal results. If a child infers a departure from its preferred outcomes—that results in a compensatory *drive* toward either self- or other-directed play behavior and a negative emotional appraisal (for more reading material on the role of appraisals in emotion theory, see e.g., Moors et al., 2013).

The levels at which these appraisals lead to a positive or negative *emotional expression* are known to vary between children (parameter *a*_7_ Oatley and Jenkins, 1996). When a child expresses positive or negative emotions, that tends to increase or decrease, respectively, its own concern for its current play behavior. Here positive emotional expressions are considered rewarding and negative expressions as discouraging a certain play behavior, as a form of self-reinforcement (*a*_1_ in Table 1).

Finally, there is continuity in the agent's behaviors: the tendency to persist in previous play behavior (play continuity; *a*_5_ in Table 1) and emotional expressions (emotional continuity; *a*_8_ in Table 1). In the literature, the notion of continuity has been described as behavioral momentum (Nevin, 1996), although that concept is defined on a somewhat larger timescale than the concept of continuity in SECONDS.

#### 2.1.2. Influencing the Other

A crucial distinction exists in SECONDS between external active states, which are observable to the other agent, and internal states, which are hidden from that agent (see Figure 1B). So only emotional expressions and play behaviors can directly impact the other agent, in the following three ways:

1. The play behavior of one member of the dyad contributes to the emergent play outcome, which determines the extent to which the concerns of the other member are realized.
2. When one member of the dyad expresses an emotion, that influences the other's concern for the play behavior associated with that emotion. Drawing parallels with self-reinforcement, social reinforcement involves positive or negative emotional expressions of one agent that are experienced by the other agent as encouraging or discouraging of associated play behavior (*a*_2_ in Table 1).
3. The agents demonstrate behavioral symmetry: the tendency to imitate the play behavior (*a*_6_ in Table 1) and emotional expressions of the other (*a*_9_ in Table 1), which has been referred to as contagion in the literature (Levy and Nail, 1993). As the multi-agent extension of continuity, the tendency toward symmetry influences the drive of the child toward a specific type of play behavior or emotional expression.

#### 2.1.3. Influencing Each Other

The emergent play outcome is only determined in the fully recurrent socio-emotional concern dynamics (SECONDS) of dyadic interaction, as we summarize in Figure 1C. Crucially, the concerns of both agents can change gradually through the accumulation of positive and negative emotional experiences associated with acting out those concerns. Therefore, while concerns shape their actions (*a*_3_ in Table 1), the results of those actions shape their concerns (*a*_1−2_ in Table 1). Such circular causality is a crucial property of complex adaptive systems, which underwrites the fact that these emergent behaviors can only be captured fully by recurrent formulations (see Figure 1C).

The full dependencies for the changes in concerns listed in Table 1 (Δ*x*_1,*t*_) show that SECONDS implements a simple form of recurrent belief-updating, in line with recent trends in cognitive neuroscience of predictive processing, reinforcement learning, and active inference (Friston et al., 2017; Sutton and Barto, 2017; Gallagher and Allen, 2018). As we described above, concerns are updated indirectly through emotional appraisals that represent the match or mismatch between session outcomes and the concerns of each agent (or, equivalently, their preferences).

### 2.2. Modeling Child Play Behavior in ASD

In dynamical modeling, we must align the features of our model with those of the target system, which in our case was a child with ASD. Our working assumption was that psychological processes of children with ASD are fundamentally similar to those of typically developing children (as defined in SECONDS), but with a number of atypical features (i.e., parameter settings) that give rise to behaviors characteristic of ASD (see also section 4.2). To apply SECONDS to children with ASD, we implemented the current clinical diagnostic criteria for this class of disorders from the DSM5 (American Psychiatric Association, 2013). A key theoretical question was which parameters of SECONDS (such as the relatedness concern) correspond to certain DSM5 criteria. Our answer therefore consists of a conceptual justification of the ways in which we implemented characteristics of ASD in SECONDS. These implementations can be viewed as a hypotheses to be explored using an agent-based model like SECONDS.

According to the DSM5, ASD is a class of disorders that share distinct impairments in social interactions and communication, typically characterized by repetitive behavior and restricted interests. We divided this definition in three aspects that correspond with the dynamic model: (1) deficits in emotional processing regarding self and other (i.e., affective communication), (2) deviant socio-emotional concerns, and (3) tendency toward repetitive behaviors. These represent only one particular realization that we derived from the ASD literature. Our specific choices here are debatable: we can use SECONDS to test them against observations and competing alternatives. For now, they simply served to demonstrate how SECONDS can be used to model ASD phenomenology:

1. Characteristic of ASD are deficits in processing information concerning one's own emotions (e.g., Hill et al., 2004) as well as the emotions of others (e.g., Bal et al., 2010). As described in the DSM5 (American Psychiatric Association, 2013), other issues are deficits in the sharing of emotions by means of facial expressions or non-verbal behavior. Using SECONDS, these deficits of emotional processing and communication were modeled as follows:
  - Hampered interpretation of actual outcomes in terms of perceived concern realization (relatively low *a*_3_). Reduced general intelligence also impairs such interpretations because they require accurate monitoring of overall session outcomes in terms of personal concerns.
  - Hampered expression of emotions following appraisal (relatively low *a*_7_).
  - Hampered adjustment of one's concerns based on the emotional expressions of the parent (relatively low *a*_2_).
2. Children with ASD often exhibit deviant socio-emotional concerns, showing more interest for inanimate objects and less interest in peers or adults compared to typically developing children (e.g., Dawson et al., 1998). Children with ASD seem to have a less strong need for relatedness with others in play than their typically developing peers. We set the initial relatedness concern of the child (*c*_*other*_) relatively low (*c*_*other*_ = *c*_*self*_ = 0.50), compared to typically developing children (whose play has been successfully modeled in SECONDS using *c*_*other*_ ≈ 0.70; Steenbeek and van Geert, 2013). Most likely, such a reduced relatedness concern is partially the result of long-term social difficulties related to the deficits in emotional processing described above. The major point of interest here was not the exact value of the parameter, but whether this value falls within the range of children with ASD.
3. As described earlier, ASD is characterized by repetitive behavior and restricted interests. Such deficits correspond to a stronger tendency for behavioral continuity in the case of ASD compared to typical development. For example, it is more difficult for children with ASD to disengage visual attention, once focused (Landry and Bryson, 2004). A second behavioral problem is concerns lack reduced mirroring of the other's behavior. Compared with typically developing children, children with ASD show deficits in imitation (for a review, see Williams et al., 2004) and joint attention (for a review, see Bruinsma et al., 2004). In SECONDS, these behavioral deficits are represented by the two non-intentional constructs of symmetry and continuity. We assumed that a child with ASD would show relatively strong continuity (relatively high *a*_5_, *a*_8_) and relatively weak symmetry (relatively low *a*_6_, *a*_9_) for both the play behaviors and emotional expressions.

### 2.3. Modeling Parental Play Behavior

We proceed to explain how we modeled similarities and differences between parent and child as they interact during free play sessions that emulate a school playground environment (see section 2.5 for a description of the observed dyad). In this setting, it was reasonable to assume the parent imitates childlike play because he or she wants the child to gain social skills that carry over to future playful interactions with peers. The simulated parent imitated natural childlike playful behavior, while also trying to realize parenting goals (as described in section 2.3.2). Indeed, such approximate symmetry derives support from observations that parents of preschool children with autism showed similar levels of overall synchrony during parent-child interactions (Siller and Sigman, 2002). There is also evidence of compensatory parental behaviors: higher levels of directiveness were observed in parents of children at high risk of developing ASD in comparison with parents with low-risk children (Wan et al., 2012, 2013).

#### 2.3.1. A Dissociation Between Playful Interaction and Displays of Parent-Child Affection

In view of our focus on modeling free play, we assumed that play interactions unfold approximately independently from displays of parent-child affection. Keeping adjustments to SECONDS to a bare minimum, we only considered other-directed behaviors that were play-related in the observational coding system. SECONDS allowed us to turn this simplification into a testable hypothesis: we tested whether such playful dyadic interactions could be modeled with SECONDS (as outlined in Figure 1), without explicitly considering parent-child displays of affection. This dissociation is theoretically plausible because relatedness and autonomy are in reality multidimensional, rather than antagonistic. We focused on the childlike play dimension of relatedness (which is the opposite of autonomy in this context) and not the affectionate (“cuddling”) dimension of relatedness. For example, a child can engage in solitary play while receiving affection from its parent. We derived that theoretical focus on play from the target system, which emulated a school playground session intended for free play specifically. Indeed, the observed dyad presented in section 2.5 exhibited a clear dissociation between the mother and child exchanging hugs and the mother and child actively playing together. For example, the child would walk around with a toy and receive a hug on the way, but it would continue playing solitarily throughout. In such instances, he maintained play autonomy by excluding his mother from the play process, despite the displays of affection. Furthermore, recent observational work by Steiner et al. (2018) suggests that, in the context of children at risk for developing ASD, parental directiveness in parent-child play interactions emerges very early on and appears to be largely independent of the child's level of socio-emotional development. It suggests that parents develop a certain style concerning their level of synchrony and directedness that is rather stable and independent of the emotional valence of a particular interaction (as also supported by Clarke-Stewart, 1973).

#### 2.3.2. Parenting Goals During Play Interactions

We departed from the original dynamic symmetry of SECONDS (as in Steenbeek and van Geert, 2005, 2008) by assuming the parent also had one-sided external control over the dynamics, which he or she could use to attain certain parenting goals. In other words, the parent had a certain power over the child—being an adult and educator—that the child did not have over the adult. The ensuing dynamics were somewhat asymmetric: the parent could maintain goals pertaining to the child's development and shape their play interactions accordingly. Note that such power does not require conscious awareness or decision-making by the parent on the level of different strategies. Parents can intuitively influence the play dynamics toward certain parenting goals, enacting more or less stable parental play strategies. Interventions could help parents to become aware of such patterns and adjust them to fit specific parenting goals.

Given the approximately childlike engagement of the parent mentioned before, we kept the basic architecture of SECONDS for the parent the same as for the child, presented in Figure 1. To leave it intact, we refrained from adjusting parental play behaviors *ad-hoc*. Instead, we devised plausible ways to attain parenting goals in SECONDS by adjusting the components and changing their causal relationships. Firstly, we made minimal adjustments that affected only the parameters of the model. For example, the parent could start with a relatively high relatedness concern, thus encouraging positive experiences of relatedness in the child. Secondly, we included extra conditions in the generative model, such as a tendency toward symmetry conditional on the child's behavior. Thirdly, we removed certain outcomes: the parent refrained from showing negative emotions when the urge arises, creating a safe environment for the child. To demonstrate modeling of implicit parental influences during play, we defined four complementary ways in which a simulated parent could work toward particular parenting goals without changing the basic architecture of SECONDS:

1. Given a parenting outcome goal, the parent can adjust his or her concerns to those of the child, allowing for scaffolding of concerns as described below (adjusting just initial concerns *c*_*self*_, *c*_*other*_, or also changes in concern *f*_1_ in Table 1).
2. The parent can selectively mirror the child's play behaviors, depending on the outcome goal (i.e., *a*_6_ in Table 1 becomes conditional on the child's behavior).
3. The parent's concerns can be satisfied selectively, introducing a motivational bias that works toward the outcome goal (i.e., *a*_3_ in Table 1 becomes conditional on play outcome).
4. The parent can use positive emotional expressions to encourage the child when it behaves in ways that are consistent with the parenting goal (i.e., adjusting *f*_4_ in Table 1).

Of course, these mechanisms are by no means exhaustive, but we could use them to simulate two parents that worked toward different outcome goals. The first parent was play-centered, in the sense that he or she focused on maximizing the amount of play during the session. The only outcome goal was playing together in order to increase the child's relatedness concern. The second parent was initiative-centered, in the sense that he or she focused on maximizing the amount of initiative-taking by the child. The main outcome goal here was to elicit initiations by the child, while playing together was a secondary outcome goal. To explain our motivation for simulating a play- and an initiative-centered parent, we provide observational background in the following section.

#### 2.3.3. Parental Imitation and Scaffolding in Play Interactions

There is a large body of research on play between parents and children with ASD that demonstrates contingent imitation of the child's play behavior increases attention and social responsiveness in children with autism (e.g., Dawson et al., 1998). El-Ghoroury and Romanczyk (1999) conducted research on dyadic play of family members with a child with ASD. They found that, in comparison to siblings, parents more often attempted to initiate play toward (their) autistic children. At the same time, autistic children more often initiated play toward their siblings than toward their parents. The results also indicated that the number of parental attempts increased with the severity of developmental delay of the children, while this pattern was not present for the siblings. This may reflect an attempt by the parents to compensate for the social deficits of their children, but the siblings elicited more initiatives from the children with ASD by giving them more space. Findings of Freeman and Kasari (2013) also indicated that imitative parental play strategies correspond with better outcomes for child-initiated play. In the context of parent-child dyadic play in general, they found that fewer commands and suggestions by the parent were associated with longer periods of joint engagement. These studies focused on imitation of play actions and the complexity of play exhibited by the child. In SECONDS, imitation can be conceived on a more abstract level as imitation by the parent of the directionality of play exhibited by the child. Self-directed play behavior of the child could be answered with self-directed play by the parent. Such imitation can be implemented in SECONDS via the parameter of behavioral symmetry, where higher symmetry of the parent will result in more imitation. The parent could also imitate the child by adjusting his or her relatedness concern to mirror the proportion of self- and other-directed behavior shown by the child.

An extension of the concept of imitation would be that of scaffolding, a term coined by Wood et al. (1976) and also indicated in the introduction. More recently, Steenbeek and van Geert (2013) implemented scaffolding by using SECONDS to model the dynamics within teacher-student dyads. The teacher aims to match the level of the student approximately, but always stays on a somewhat higher level located within what Vygotsky and Cole (1978) called the zone of proximal development. The teacher tries to maintain an optimal scaffolding distance for learning. Research has shown that for typically developing children scaffolding by the parents is essential for development (e.g., Hammond et al., 2012). Given their learning deficits, the bandwidth of effective scaffolding would be expected to be relatively small for children with ASD. Still, even without any training, mothers of children with ASD have been observed to apply verbal scaffolding that was appropriate to the developmental level of their children (Konstantareas et al., 1988). In research by Pierucci (2014), mothers of young children with ASD were taught to apply scaffolding techniques more effectively in parent-child play, which was found to increase social engagement of these children.

In the context of SECONDS, we conceptualized scaffolding in terms of the relatedness concerns. The parent could keep their relatedness concern at a level just slightly higher than that of the child. Since the real-time level of the child's relatedness concern is not directly observable, the parent would need to make an estimate based on the child's previous and current behaviors. The parent could then aim to find the optimal distance for scaffolding the relatedness concern. However, such optimization is not straightforward if there are multiple conflicting outcome goals. To demonstrate this point, we defined two parental outcome goals (hinted at in section 1). The first is playing together with the child (“give a man a fish”) and the second is helping the child to practice play initiation (“teach him to catch a fish”). Unfortunately, playing together and play initiation are two learning outcomes that compete with each other in this setting. For example, if the parent can maximize the amount of joint play by initiating it (i.e., a large scaffolding distance in the relatedness concern), but that approach deprives the child of opportunities to practice the initiation of play. We now proceed to describe the precise adjustments for both the play- and initiative-centered parent, while highlighting their differences.

#### 2.3.4. The Play-Centered Parent

The play-centered parent maximized the time spent playing together through the following four mechanisms (introduced in the previous section):

1. Initially, this parent was much more concerned with relatedness than the child: *p*_*other, initial*_ = 0.65, *c*_*other, initial*_ = 0.50, a large scaffolding distance compared to the initiative-centered parent. Over the course of each session, this parent aimed to scaffold the child's concerns by being more concerned with relatedness: *p*_*other*_ > *c*_*other, initial*_ = 0.50. This lower bound was based on the child's initial concern, because real-time changes are harder to estimate for the parent (see Figure 1B for the distinction between hidden and observable components of each agent). In the simulations, we confirmed that this parent was more concerned with relatedness than the child throughout each session.
2. This parent exhibited strong selective symmetry toward playing together: he or she tended to imitate the child more strongly in other-directed play than in self-directed play (i.e., *a*_6_ was conditional on the child's behavior).
3. This parent encouraged the child's other-directed behavior through positive emotional expressions and implicitly discouraged the child's self-directed behavior by avoiding positive emotional expressions during such behavior. He or she refrained from showing negative emotions during all interactions. We implemented these adjustments by changing *f*_4_ in Table 1.
4. Satiation of this parent's concerns took much longer for relatedness than for autonomy (i.e., *a*_3_ in Table 1 was conditional on play outcomes), introducing a bias toward other-directed play.

#### 2.3.5. The Initiative-Centered Parent

The initiative-centered parent maximized the time spent playing together as a result of the child's initiations, while maximizing joint play was only of secondary importance. This parent tended more toward imitation of the child and was less selective than the play-centered parent, increasing the number of opportunities for the child to elicit play. The four mechanisms were set as follows:

1. Initially, this parent was slightly more concerned with relatedness than the child: *p*_*other, initial*_ = 0.55, *c*_*other, initial*_ = 0.50, a small scaffolding distance compared to the play-centered parent. Over the course of the session, the parent aimed to scaffold the child's concerns by always being slightly more concerned with relatedness: *c*_*other*_ + 0.01 < *p*_*other*_ < *c*_*other*_ + 0.15. This parent intended to maintain a scaffolding distance that provides an optimal balance between both outcome goals: eliciting initiations of the child and playing together.
2. This parent exhibited relatively weak selective symmetry, with only a slight preference toward mirroring other-directed play behaviors. Their overall tendency toward symmetry was fairly strong, such that this parent imitates the child.
3. The selective use of positive emotional expressions was limited and they do not discourage playing alone by withholding positive emotional expressions. Being an adult, this parent did refrain from showing negative emotions during play.
4. Satiation of this parent's concerns took slightly longer for relatedness than for autonomy, introducing a slight bias toward other-directed play.

In this way, two different parents were modeled to be either play-centered or initiative-centered, representing different outcome goals. The parameter settings for the child with ASD were identical in both dyads. Naturally, the simulated dyads are only two of the many possibilities, and therefore they were used for exploratory purposes, and should in no way be considered exhaustive. Parents and children with ASD show large inter-individual differences, such that we expected to observe large differences between these two simulated dyads, but also between both simulated dyads and a real parent-child dyad. We sought to determine whether the differences between the two simulated dyads conform to our expectations (section 7) and whether the simulated parents constitute plausible representations of a real parent-child dyad in the context of ASD, based on an observed parent-child dyad (section 2.5).

### 2.4. Comparison Between Simulated Dyads With Play- and Initiative-Centered Parents

We compared the two parent-child dyads simulated in SECONDS across 2,000 simulation sessions on the distributions of seven variables that summarized session outcomes concerning play and initiation. Six of these summary variables represented the total time allocated to play and initiation^7^. Proportional (dimensionless) time allocation was chosen for comparison with observations because it is more robust than frequency measurements, which depend on the time resolution of the observations and simulations. The expectations for the differences between the two parents on all seven variables are summarized together in Table 2.

**Table 2:** Comparison of the two simulated parent-child dyads in terms of our expectations for the relative ordering of the medians, across 2,000 simulated play sessions.

	**Expectations**	**Simulated dyads**		
		**Play**		**Initiative**
*variable*	Ordering of medians	Median		Median
	(**play** … **initiative**)	[95% CI]		[95% CI]
*together*	(**play** > **initiative**)	40.9%	>	34.2%
		[40.0–42.7%]		[33.3–35.1%]
*alone*	(**play** < **initiative**)	4.0%	<	18.2%
		[3.6–4.0%]		[17.3–19.1%]
*attempt*_*child*_	(**play** < **initiative**)	0.9%	<	4.4%
		[0.4–0.9%]		[4.0–4.4%]
*success*_*child*_	(**play** < **initiative**)	0.0%	<	6.7%
		[0.0–0.0%]		[6.2–7.6%]
*c*_*inv*_	(**play** > **initiative**)	0.567	>	0.520
		[0.564–0.571]		[0.518–0.522]
*attempt*_*parent*_	(**play** > **initiative**)	47.6%	>	29.8%
		[46.2–48.9%]		[28.4–31.1%]
*success*_*parent*_	(**play** > **initiative**)	34.2%	>	20.4%
		[32.9–35.1%]		[19.6–21.3%]
**Play**: dyad with play-centered parent				
**initiative**: dyad with initiative-centered parent				

*We also provide 95% confidence intervals (CI) for these medians based on the sample size (n = 2,000)*.

Firstly, for common play events we measured the proportion of time spent during each session: both parent and child playing *together* and both playing *alone*. Obviously, we expected the play-centered parent to spend more time playing *together* with the child than the initiative-centered parent. Both playing *alone* depended strongly on the tendency of the parent (who has a stronger relatedness concern) to imitate the child when it chose to play alone. Such space was expected to provide the child more opportunities to initiate play. Therefore, we expected the initiative-centered parent to allocate more time to both playing *alone* than the play-centered parent.

Secondly, for each member of the dyad we also measured attempts to initiate play and joint play resulting from these attempts in each session. In SECONDS, we defined attempts at initiation by the child (*attempt*_*child*_) or parent (*attempt*_*parent*_) as instances where one engaged in other-directed play behavior (i.e., communicating the desire to play together), while the other engaged in self-directed play behavior. A successful play initiation occurred when the play invitation of child or parent was followed by playing *together* for some time. The entire duration of that initiated joint play was added to *success*_*child*_ or *success*_*parent*_, depending on who took the initiative. In this way, we compared play behaviors and initiations of the child and its parent. Attempts at initiating play by the child showed how much space was given to the child to engage in these attempts. Successful initiation of play by the child indicated how much experience they gathered in mastering this pivotal response class described before. We expected the child to show more attempts at playing together and more play resulting from these attempts for the initiative-centered parent. We expected the play-centered parent to show more attempts at playing together and more play resulting from these attempts.

Thirdly, we also compared the relatedness concern of the child at the final time step of the simulations in SECONDS (*c*_*other, final*_), which summarized motivational changes in the child with respect to the initial situation (*c*_*other, initial*_ = 0.50). Since experiences of playing *together* tend to increase one's relatedness concern, we expected *c*_*other, final*_ to be higher for the play-centered parent than for the initiative-centered parent.

Crucially, resulting play outcomes emerged from the dynamic coupling between the behaviors and emotional expressions of the child and its parent (see Figure 1C). These outcomes were not linear or additive outcomes of the parameters, and it was necessary to run simulations with SECONDS for both play- and initiative-centered parents to test whether the expected outcomes would be obtained.

### 2.5. Case-Based Model Validation Using Real-Time Play Data of a Mother-Child Dyad

For model validation, we employed a single in-depth case study of free play between a child with ASD and its mother. Each 1-h session consisted of phases simulating different activities during a school day, resulting in 29 video-recorded episodes of free play, lasting about 15 min each. During these episodes, the mother and child freely engaged in play, using a variety of available toys. No learning goal was formulated for this phase. This single real dyad (observed for 29 sessions) was compared with the two virtual dyads we simulated using SECONDS (for 2,000 sessions each) on the six types of events described in section 7: both playing *together* and *alone*, play initiation attempts by the parent and child (*attempt*_*child*_, *attempt*_*parent*_), joint play resulting from attempts of the parent and child (*success*_*child*_, *success*_*parent*_).

We realize an *n* = 1-study like ours will be raising some eyebrows for those accustomed to standard research practices in psychology. However, complexity-based approaches actually favor model validation on a case-by-case basis over group statistics (Molenaar and Campbell, 2009). In the Discussion (section 4), we provide an in-depth justification of this methodological choice. For now, we note that a single case study is a valid and informative starting point if it is explanatory^8^. Our study can provide reliable information on a whole class of cases because it met the following three specific criteria for explanatory case studies (Flyvbjerg, 2006; Yin, 2009):

1. It answers a “how” question: we sought to model the mechanism that explains play dynamics between a child with ASD and its parent.
2. It examines a contemporary phenomenon in context: the social deficits exhibited by children with ASD are well-documented today (i.e., contemporary) and we considered interactions with its own caregiver (i.e., in context) in helping the child to habituate to the pattern of schooling in general.
3. There is no experimental control over the explained phenomenon: the play sessions were structured as a school break consisting of free play without specific goals.

The amount of data that was necessary to make even this single-case comparison was overwhelming: time-series of over 26,000 data points were collected for the real dyad. Of course, we do not claim that a single case is sufficient: case-based model validation is a cyclical process. Collecting more such in-depth cases in the future can reveal patterns across sessions and within the population of children with ASD, revealing inter-individual as well as intra-individual differences.

Observational data were gathered from free play sessions occurring in the context of an intervention study (unrelated to the model at hand, see Steenbeek et al., 2017) in which a 9-year-old boy diagnosed with severe (classical) ASD practiced school activities together with his mother in biweekly sessions over 18 months. He was able to verbalize some of his intentions, although it cost him significant amounts of effort. Due to the severity of his symptoms, the boy was exempt from any form of education (both regular and special) by the Dutch government at the age of five. His parents have been more or less obliged to give him home-schooling, which they have been doing partly in the context of the Autism Project, a collaboration between researchers from the University of Groningen and the Hanze University of Applied Sciences.

Free play sessions were about 15 min each (with some variation). Coding was event-based and exhaustive. One session out of 29 was used for reliability training between two independent observers, which led to a substantial inter-rater reliability of κ = 0.68 on subsequently coded sessions, corresponding with 89 percent inter-observer agreement. These video fragments were first coded in terms of whether the parent and child were playing together or not. Periods of not playing together were then coded in terms of whether both parent and child were playing alone or whether one of them was trying to initiate play. To maximize inter-observer reliability, the operational definition of play initiation was taken to be a verbalized communication of a desire to play together. Finally, the resulting time-series were used to measure the percentages of time allocated to the six types of events listed in section 7: both playing *together* and both *alone*, attempts to initiate play by the child and its parent (*attempt*_*child*_, *attempt*_*parent*_), and play resulting from initiations by the child and its parent (*success*_*child*_, *success*_*parent*_). Video recordings were not detailed enough to track emotional expressions with high reliability, so we have left overt emotional expressions out of the analyses. In translating the simulation output to real observations, we assumed an initiation attempt would be verbalized when it persisted for at least two simulation time steps. This correction accounted for the fact that the observational coding system was limited to verbalized attempts, while SECONDS included both verbal and non-verbal other-directed play behaviors.

For both simulated dyads and the observed dyad, we compared their smoothed distributions (i.e., kernel density estimates) across sessions on these variables. Furthermore, we quantified the fit between each distribution and the observed dyad in terms of the Kullback-Leibler divergence (*KLD*), a well-established information-theoretic measure of statistical divergence (Kullback and Leibler, 1951). *KLD* is given in information units of *nats* (the equivalence of bits, but based on powers of *e*): the smaller *KLD*, the better the observed fit.

For a baseline comparison, we used beta distributions—a common tool in Bayesian statistics for describing probability distributions of proportions (such as our variables). A naive observer who knows nothing about the system at hand – except that it has four possible states—could use the reasonable starting point of beta distributions with an expectation value of 25 percent for each of the four different states of the system: (1) *together*, (2) *alone*, (3) *attempt*_*child*_, and (4) *attempt*_*parent*_. Naively speaking, playing together would be attributed equally to *success*_*child*_ and *success*_*parent*_, each with a long-term expectation value of 12.5 percent. Further technical details are provided in a footnote^9^. These reference distributions served a twofold function. First of all, they allowed us to check whether our simulated dyads indeed exhibited similarities to the types of behaviors we would typically expect from the target system. Secondly, these reference distributions provided us with null hypotheses that allowed us to test whether SECONDS actually helped to provide a better fit with the observed distributions. That step allowed for an interpretation of *KLD* values in terms of more common statistical methods. We calculated the *p*-values of the set of reference distributions for both simulated dyads by transforming *KLD* values back to probabilities and normalizing: *p*_0_ = *e*^*KLD*_0_^/(*e*^*KLD*_0_^ + *e*^*KLD*_*model*_^), where *p*_0_ is the likelihood of the reference distributions (with *KLD*_0_) given the observations and an alternative set of distributions generated by SECONDS (with *KLD*_*model*_).

## 3. Results

### 3.1. Comparison Between the Two Simulated Parents-Child Dyads

**Figure 2:**
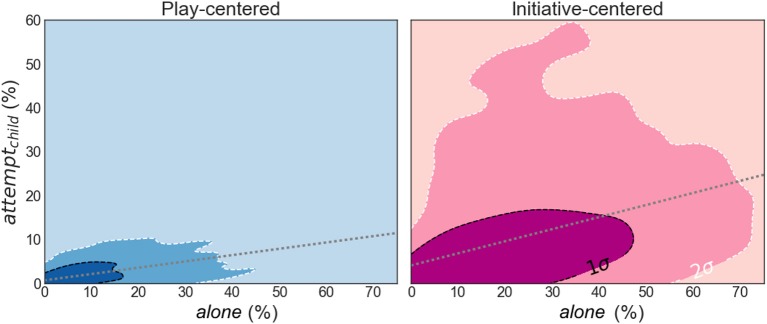
A comparison between the two dyads with either a play-centered parent (blue, left panel) or an initiative-centered parent (pink, right panel): density plots across 2,000 simulated sessions of the time spent both playing *alone* (percentage of session time; horizontal axis) versus attempts to initiate play by the child (*attempt*_*child*_; percentage of session time; vertical axis). High density regions are darker than low density regions. 1- and 2-σ confidence intervals are indicated with dashed lines: 68 percent of the sessions fall within the black contours; 95 percent of the sessions fall within the white contours. Within each dyad, the more time is spent both playing alone, the more time is typically spent initiating play by the child – as we illustrate with simple linear regression (gray dotted lines; play-centered parent: *attempt*_*child*_ = 0.62% + 0.14·*alone*, *R*^2^ = 37%; initiative-centered parent: *attempt*_*child*_ = 4.0% + 0.28·*alone*, *R*^2^ = 13%). As expected, this comparison shows that the play-centered parent allows for less solitary play and fewer attempts to initiate play by the child overall, compared to the initiative-centered parent.

The differences between the two simulated parents corresponded with our expectations (as shown in Table 2). The most important difference between these two simulated parents was apparent in the distribution across simulations of the time attributed to *alone* and to *attempt*_*child*_ (as shown in Figure 2). Here *attempt*_*child*_ is a percentage of the time that remains after *alone* is accounted for. Longer periods of solitary play were related to more initiative-taking by the child, as shown by linear regression analyses (play-centered parent: *attempt*_*child*_ = 0.62% + 0.14·*alone*, *R*^2^ = 37%; initiative-centered parent: *attempt*_*child*_ = 4.0% + 0.28·*alone*, *R*^2^ = 13%). For the play-centered parent, *alone* and *attempt*_*child*_ were close to zero most of the time, but the few higher values of *alone* were also related to higher values of *attempt*_*child*_. As expected, the initiative-centered parent was more effective than the play-centered parent in eliciting play initiation attempts by the child.

### 3.2. Comparison Between Simulated and Observed Parent-Child Dyads

**Figure 3:**
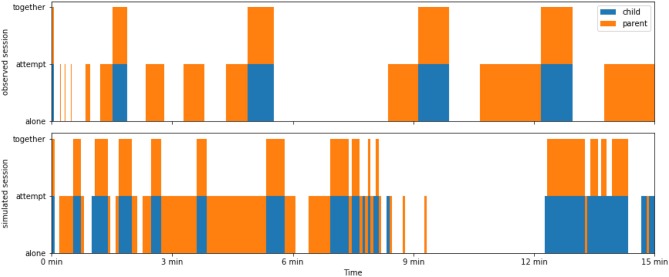
An illustrative comparison of the play dynamics of parent and child as they unfold over the course of 15 min in one example of an observed session (upper panel) and one of a session simulated with SECONDS (lower panel; involving the initiative-centered parent). These stacking plots indicate with colored areas when the parent (orange) and child (blue) are engaging in other-directed play behavior. The absence of blocks indicates they are both playing *alone*, single blocks (either orange or blue) indicate one of them is attempting to initiate play (*attempt*_*child*_ or *attempt*_*parent*_), and two stacked blocks (orange *and* blue) indicate they are playing *together*. The qualitative similarities between these panels are striking. However, both the observed dyad and the simulated dyads exhibit large variability across sessions, so a comparison across sessions as shown in Figure 4 is crucial.

Our data represent three different parent-child dyads in the context of ASD (two virtual, and one real). As for real parent-child dyads, we expected to find both similarities and differences. In Figure 3, we illustrate the ensuing dynamics by comparing an observed and simulated play session (with the initiative-centered parent). While correspondence between these example sessions is obvious, both the real dyad and simulated dyads showed large variation across sessions. Therefore, we also examined distributions across sessions in order to evaluate the plausibility of the simulated dyads.

**Figure 4:**
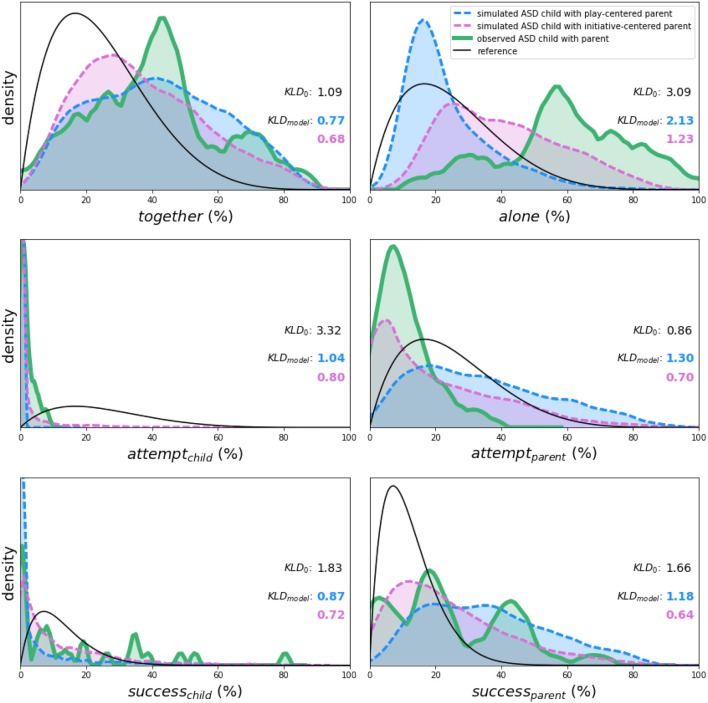
Comparison of kernel density plots (similar to histograms) on all six variables between two simulated parent-child dyads (blue and pink dashed lines), one observed parent-child dyad (green solid lines) across play sessions in the context of ASD, and reference beta distributions (black line). Simulated distributions summarize 900,000+ time series data points generated by SECONDS – 225 time points over 2,000 sessions for each of the two dyads: a play-centered parent (blue) and an initiative-centered parent (pink). Observational distributions summarize over 26,000 time series data points gathered from one observed dyad (29 sessions; green). A qualitative, visual comparison of the distributions for simulated and observed dyads suggests surprisingly strong correspondences. We quantify the fit between each distribution and the observed dyad in terms of the Kullback-Leibler divergence (*KLD*; in information units of nats). The lower *KLD*, the better the fit. Most conveniently, *KLD* values are additive across variables, allowing us to estimate total model fit through summation. The simulated dyads have total *KLD*_*model*_ of 7.28 and 4.76 *nats* for the play- and initiative-centered parents, respectively. Therefore, they outperform the reference distributions, which have a total *KLD*_0_ of 11.83 *nats*, and the initiative-centered parent provides the best fit overall.

Across sessions, simulated and observed dyads exhibited qualitative similarities as well as differences (as shown in Figure 4). This observation is itself noteworthy—these two virtual dyads simulated in SECONDS showed a plausible degree of similarity to an actual parent-child dyad, especially given the idiosyncrasies associated with ASD (e.g., Vivanti et al., 2014; Hahamy et al., 2015). Moving beyond a subjective qualitative comparison, we also conducted a statistical comparison between simulated and observed distributions. Most conveniently, *KLD* values are additive across variables. For the reference distributions, we obtained total *KLD*_0_ = 11.83 *nats*. For the simulated dyads with play- and initiative-centered parents, we obtained total *KLD*_*model*_ = 7.28 and 4.76 *nats*, respectively. These values indicate that both dyads with play- and initiative centered parents showed a smaller divergence from observations than the reference distributions. Comparing the reference *KLD*_0_ with *KLD*_*model*_ as described in Section 9, we obtained *p*_0_ = 0.046 and 0.0048 in favor of the simulated dyads with play- and initiative-centered parents, respectively^10^. We calculated these *p*-values mostly for the benefit of the reader, so we did not decide on any particular α-value of significance. In any case, we can conclude that (1) both simulated dyads provided a better fit to observations than the reference null distributions and (2) among the simulated dyads, the one with an initiative-centered parent provided a better fit than the one with a play-centered parent.

## 4. Discussion

We successfully extended SECONDS—an agent-based model of socio-emotional concern dynamics developed for child-peer play—to the case of parent-child play in ASD. With our settings and minor adjustments, SECONDS produced session data for two parent-child dyads that (1) showed agreement with our theoretical expectations in an internal model comparison—see section 7—and (2) produced play and initiation time distributions with a plausible degree of similarity to observational data of a real mother-child dyad with ASD—see section 2.5. We found strong, non-trivial correspondence between the two simulated dyads and the single observed dyad (as compared to null reference distributions). Overall, the overlap is strong enough to show that our two simulated dyads are plausible representatives of what we aimed to model. In section 4.1, we explain what plausibility means in the context of case-based validation of process-oriented models and in section 4.2 we discuss the generalizability of our results.

Our primary modeling goal was to construct a psychologically informed agent-based model that could generate plausible real-time descriptions of play between a child with ASD and its parent. The scientific heavy-lifting was done on the theoretical level, since few (if any) such models have been developed before. Since observational validation would need to happen on a case-by-case basis (as we explain in section 4.1), we measured whether time series of interactions within a particular observed parent-child dyad would fall within a plausible range of correspondence with two simulated dyads. Our research provides a proof of principle for dynamical modeling of real-time decision-making in child-parent dyadic play in ASD^11^.

### 4.1. The Case for Case-Based Model Validation

Our argument follows the consensus view among complexity-oriented researchers in psychology that process-oriented models for psychological phenomena can usually not be validated on the basis of inter-individual variability (Toomela, 2007; Byrne and Ragin, 2009; Castellani and Hafferty, 2009). In section 1.1, we explained that aggregated data can only be used for processes under the statistical assumption of ergodicity, which is often violated in psychological phenomena (Molenaar, 2004), and even more so in heterogeneous conditions like ASD. If researchers instead accumulate single cases, that is likely to lead to insightful clustering that reveals relevant similarities and differences (as shown by Castellani and Hafferty, 2009). At least one case study like ours is needed to decide whether that would be a worthwhile endeavor^12^. Presenting our simulations without a comparison to a particular case would diminish the relevance of our publication as a proof of principle^13^. To the best of our knowledge, the extant literature does not provide time-serial data on parent-child play in ASD. Our report provides a starting point for other investigators who wish to study real-time interactions between adults and children with ASD, or other types of dyads^14^.

As surprising as it may sound, we can draw robust conclusions on the plausibility of our simulated dyads from this single parent-child dyad. The child in our study was representative of children with severe ASD in a qualitative sense, which does not require correspondence with summary statistics of all children with severe ASD. In process-oriented modeling, plausible representation means that a second pair from the same population (parent-child dyads with ASD) would typically differ from the first pair within approximately the same range as our simulations. Given the detail of our observational data (over 26,000 data points) and the heterogeneity of ASD, it is surprising that our attempt showed such strong correspondence. One line of work would involve fitting the parameters of SECONDS until they produce the exact behaviors of the observed dyad.

### 4.2. Generalizability: Processes Vs. Parameters

SECONDS was developed as a generic model which could be applied to different cases by using different values for a generic set of parameters (such as *c*_*other, self*_ and *a*_1−9_ in Table 1). We demonstrated that the conceptual makeup of SECONDS can be generalized (1) to children with severe ASD and (2) to parent-child dyads. The first result suggests that children with severe ASD engage in play based on psychological processes that are fundamentally similar to those of typically developing children (i.e., socio-emotional concern dynamics), albeit operating under different parameters. This result provides support for the increasingly common approach of modeling ASD and other psychiatric conditions as atypical expressions of general neurocognitive mechanisms (e.g., Constant et al., 2018). The second result suggests that free play between parent and child can be modeled as if the parent imitates childlike behavior. Since all kinds of specifics of the parent-child relationship can have considerable influence on the dynamics of play, this finding is non-trivial.

In evaluating the generalizability of SECONDS, there are two levels at play: (1) the psychological processes represented by its computational architecture and (2) the specific range of parameter settings used for modeling populations of interest. In complexity-oriented approaches, generalizability refers primarily to the first level: it requires theoretical justification and incremental observational validation, both of which we present in this paper. Generalizability refers only secondarily to the second level, which requires empirical work beyond what we present here. In principle, every individual dyad from a population can be represented by a dyad-specific parameter set (such as *c*_*other, self*_, *a*_1−9_ in Table 1). Determining which range of parameter settings provide the best explanation for the population of parent-child dyads with ASD is an empirical matter that inevitably requires studying more cases.

### 4.3. Disentangling Causal Multiplicities With Agent-Based Models

Understanding the causal structure of underlying processes is important for improving predictions of socio-emotional concern dynamics in individual children with ASD. A higher level of specificity can help to develop personalized treatments that maximize therapeutic benefits for each individual. ASD symptoms involve a multiplicity of causal factors due to the reciprocal relationships between social, cognitive, and affective processes. When multiple sets of parameters explain the same data, the corresponding models are called *degenerate*. In section 2.2, we discussed how reduced processing of emotional information and a low relatedness concern both explain reduced other-directed play behaviors. Since these two issues are interactive, we assumed both are typically present in children with severe ASD. There are other such degeneracies that reflect the idiosyncratic nature of ASD (Vivanti et al., 2014; Hahamy et al., 2015). For example, a lower relatedness concern and language deficits can both reduce a child's verbalized attempts to initiate play. In our observational setting the parent often waited for an explicit verbal invitation from her son for educational purposes, which made it more difficult for the child to initiate play. During the coding of the data, such blockage of the child's behavior was observed multiple times and his subsequent responses appeared to depend on the strength of his desire to play together. Communication deficits related to ASD can be taken into account more explicitly in future versions of SECONDS by incorporating both a non-verbal and verbal channel of interaction (see section 4.4).

The observation that similar behaviors may result from a multiplicity of causes is a strength, rather than a weakness of SECONDS and comparable agent-based models. Complex adaptive systems (like humans) can meet external and internal demands most efficiently when they possess the flexibility to respond in multiple ways to any given problem (e.g., Den Hartigh et al., 2016). If we implement process-oriented explanations in agent-based models (such as SECONDS), we can interrogate these models to produce specific predictions on intra-individual variability, which population-oriented models are not capable of. For example, we can predict the ensuing temporal dynamics for an individual child given a particular treatment option. This specificity helps (1) researchers to tease apart the degeneracies mentioned above and (2) clinicians to provide effective treatments that are tailor-made for the individual child.

### 4.4. Recommendations for Further Development

Our observations were limited to verbalized attempts at initiation to maximize inter-observer reliability, while SECONDS generated other-directed play behaviors in general. Gestures are often used as communication by children, and even more so by those on the ASD spectrum who have language impairments (true for the child in our study). As mentioned in section 2.5, we translated the simulation output to observations by assuming that attempts at initiation were verbalized when they persisted over two successive time steps in the simulations. That tension between verbal and non-verbal communication could be resolved by including non-verbal communication in the observational coding system. As a result, less time would be categorized as both playing *alone*—likely producing better correspondence with SECONDS (as outlined in Figure 4). Although such changes would introduce higher observer ambiguity, that same ambiguity also more closely resembles the world as experienced by the members of the dyad. A more interesting option would be to incorporate speech directly in SECONDS in terms of two communication channels: non-verbal and verbal, where a strong drive to play together can motivate a verbally impaired child to make the additional effort to speak. The relatively clear verbal channel would typically require a stronger drive than the relatively noisy non-verbal channel. The level of language skill of a child then sets the amount of effort required for the verbal channel, while the child's relatedness concern will influence the amount of effort invested. Verbal and non-verbal communication are characterized by different levels of ambiguity, which could be quantified directly using Bayesian statistics (as in predictive processing; Clark, 2013).

More generally, SECONDS lends itself well for integration with state-of-the-art Bayesian accounts of neurocognitive function and predictive processing (Clark, 2013) because it already implements a form of belief-updating for concerns (Δ*x*_1, *t*_ in Table 1). Furthermore, Ridderinkhof (2017) presented a conceptual integration of predictive processing and the emotion theory on which SECONDS was based (Frijda, 1986). Given recent simulation work on emotions (Allen et al., 2019; Smith et al., 2019), we believe it to be especially promising to integrate SECONDS with *active inference*—a complexity-oriented Bayesian framework (Friston et al., 2017; Hesp et al., 2019). Active inference can be used to model emergent functions and multi-scale integration (Ramstead et al., 2019), an adequate framework for socio-emotional development as in SECONDS. For example, Smith et al. (2019) presented an active inference model of emergent emotional state inference and emotion concept learning. As mentioned, a more explicitly inferential (i.e., Bayesian) formulation of SECONDS would allow for modeling the different degrees of uncertainty associated with verbal and non-verbal communication channels. It could also be used to model recent theorizing on the neurocognitive underpinnings of ASD (Parr et al., 2018). For example, researchers (e.g., Constant et al., 2018) have argued that overly precise (implicit) expectations could provide a unified way to model a wide array of disparate ASD symptoms, such as (1) repetitive behaviors, (2) increased distress when expectations are being violated, and (3) reduced integration of new information—especially under large uncertainties typical of social interaction. By modeling Bayesian inference (currently implicit in SECONDS), we can model the emergent effects of such overly precise expectations on dyadic interaction.

For our study, the parameters of SECONDS were adjusted according to our expectations as outlined in section 2. Fitting parameters directly to many specific cases from a population would be more challenging numerically (and observationally!) but would provide more robust tests of SECONDS. Such model fitting allows for the development of diagnostic tools (e.g., questionnaires, short tasks) that estimate the parameter values for individual subjects (an approach called computational phenotyping; e.g., Friston et al., 2017). A dyad could then first be tested to measure these parameters, after which the outcomes of real interactions can be compared with simulated outcomes. Subsequently, such knowledge can be employed in the design of personalized behavioral strategies for parents of children with ASD. Measuring the parameter groups repeatedly at the end of each session would also allow for testing predictions concerning long-term changes in these parameters. Hypothesized mechanisms for such long-term changes can be directly implemented in future versions of SECONDS. For example, by taking the final values of the relatedness concern *c*_*other, final*_, we can simulate changes in relatedness concern across the sessions. This has been done in a simulation of constructive dyadic play over 6 repeated sessions, thus modeling medium-term changes in constructive play parameters (see Steenbeek et al., 2014).

### 4.5. Conclusions

We demonstrated how SECONDS—a validated agent-based model of socio-emotional concern dynamics (Steenbeek and van Geert, 2005, 2008)—can be applied to real-time playful interactions between parent and child in the context of an idiosyncratic developmental disorder like ASD. Because SECONDS was originally derived from verbal psychological theories of behavior, our translational research increases the relevance of theory to empirical and clinical work in developmental psychology. SECONDS can help to disentangle conceptual degeneracies in the etiology of ASD. Within SECONDS, variations between simulated parents corresponded with expectations derived from previous literature (as discussed in section 7). The two simulated parent-child dyads showed better correspondence with the observed dyad than reference null distributions (as discussed in section 2.5). Based on these results, we conclude that the two simulated dyads are plausible representatives of parent-child dyads in the context of ASD. Given the strong correspondence, it is likely that remaining differences between these simulations and observations can be accounted for by direct fitting of parameters, increasing the number of observed dyads, and moving in future directions of SECONDS (outlined in section 4.4). Fitting more cases will allow us to further establish and improve the predictive value of SECONDS in characterizing the dynamics of such playful interactions. Although much exploration is left to be done, we conclude that our work provides a proof of principle that a dynamical model of play between typically developing children can indeed be adjusted to account realistically for another type of dyad, such as that of play between a parent and a child with ASD. Thanks to the virtual environment of SECONDS, our investigation opens the door toward the use of agent-based modeling as a cost-effective and ethical way to design and test new therapeutic interventions that stimulate the socio-emotional development of ASD children.

## Ethics Statement

This study was carried out in accordance with the recommendations of Ethical Committee Psychology, University of Groningen, the Netherlands with written informed consent from the caregivers of the subject. The caregivers gave written informed consent in accordance with the Declaration of Helsinki. The protocol was approved by the Ethical Committee Psychology, University of Groningen, the Netherlands.

## Author Contributions

CH rewrote the child-play model in Python in order to allow for flexibility, incorporated the changes in the model for the ASD parent-child dyad, ran the simulations, coded the observational data, supervised the inter-observer reliability measurements, compared and interpreted the correspondences between simulations and observation, produced the figures, and wrote the first draft. HS and PvG provided the source code and background to the original child-play model and worked to improve the first draft. HS provided supervision and theoretical background to the developmental aspects of ASD and set up the collaboration that collected the video material of the real ASD parent-child dyad. PvG provided theoretical background to the complexity approach and dynamical modeling.

### Conflict of Interest Statement

The authors declare that the research was conducted in the absence of any commercial or financial relationships that could be construed as a potential conflict of interest.

## Footnotes

^1^Alternatively, we can illustrate how group statistics occlude crucial information on individual events with the following silly parable: “A man tries to shoot a pigeon, but he hits a tree to the left instead. After a while, the bird returns to its favorite branch, but the man's second shot veers off to the right. After concluding that the bird must be dead on average, the man returns home satisfied.” The point of this parable is that central tendencies, with the average taken as typical example, can lead to paradoxical or meaningless conclusions when applied to temporal variability, i.e., to processes.

^2^Concerns specify the system's attractor states, toward which it tends to gravitate.

^3^Behaviors are the state of the agent in action space.

^4^Later on, when we introduce the parental play strategy, we dissociate these two concerns partially through selective satiation.

^5^Concern realization characterizes the match between the system's current state and its attractor state. More advanced formulations could implement allostatic concern realization: locally departing from one's concerns to attain concern realization on longer time scales (e.g., a child isolating itself intentionally to attract the attention of its parent).

^6^Drives and appraisals represent the pull on the agent toward particular action states.

^7^These six externally observable variables were also measured for comparison with the real mother-child dyad.

^8^It is intentionally called an “explanatory” case study (not “exploratory”), since we aimed to explain the studied case.

^9^The relevant probability distribution for a dynamic system that has four possible states is a 4-dimensional Dirichlet distribution, producing four marginal beta distributions. The beta distribution is a special case of the Dirichlet distribution. It has been used for modeling behaviors of random variables on finite intervals across disciplines. In Bayesian statistics, the beta distribution is commonly used to characterize one's prior knowledge about a probability or a proportion. In our case, we used it to specify the null hypotheses on the interval between 0 and 100 percent. The beta distribution has two parameters α and β, which we set such that the expectation value of four variables was 25 percent on the long term (α = 2 and β = 6). Furthermore, playing *together* was sub-divided into *success*_*child*_ and *success*_*parent*_, such that the latter two variables had a baseline long-term expectation value of 12.5 percent (obtained using α = 2 and β = 14). In absence of additional information, the naive observer would also need to guess the variance (set by the proportion between α and β).

^10^As an additional check, we also performed an analysis using a different (though much less reasonable) null hypothesis: a uniform probability distribution. That yielded the same result: namely that SECONDS produces a better fit by a large margin.

^11^Generating sample-based statistics, such as population averages, can be a secondary aim of agent-based modeling. Such population statistics result from the capacity of SECONDS to generate empirically valid case-specific time series for a range of parameter values that simulate dyads from a particular population. However, that secondary aim can only be pursued after the first aim has been achieved: to demonstrate that SECONDS is capable of generating time series descriptions that come sufficiently close to real parent-child dyads in ASD to warrant further work. It is this first aim that we pursued, in the form of taking a step in the presentation, simulation, and empirical validation of SECONDS.

^12^Gathering over 26,000 data points on this single dyad was extremely labor intensive and only this single case was available for such intensive observation—as part of a pilot study preparing for a broader group of ASD children, aimed at improving the scaffolding dynamics in school contexts.

^13^Of course, the observed dyad should be randomly selected. It should not be selected as a function of arbitrary correspondence with an already existing model simulation.

^14^Given that ASD has been so extensively studied in the scientific community, we would encourage researchers to email the corresponding author (c.hesp@uva.nl) in case they have access to data that could be used for similar modeling work.
